# Dr. Adams, on Morbid Poisons

**Published:** 1801-06

**Authors:** Joseph Adams

**Affiliations:** Madeira


### To Dr. WILLAN.

My dear Sir,

THOUGH I have already anfwered your obliging communication relative to the cow-pock, yet I cannot lofe the prefent opportunity of again enquiring in what particulars "this fingular complaint, (to ufe your own words) illuftrates my ideas even better than the fmall-pox?" Pray give me every information you can; and be affured I will not be backward in following up every experiment my fituation here will admit. Dr. Pearfon's threads were fo long in their paffage, that I was fearful of hazarding the reputation of the new practice on fuch materials. I have, however, procured a cow to make the firft effay on; and if the experiment is completed before the veffel fails, will acquaint you with the refult.

In the mean time, it is much my wifh to refer you to a paper of Dr. Haygarth's, in No. XIII. (Vol. iii. page 198) of the Medical Journal, entitled, "Of Fever from the Venereal Poifon." You need only perufe the paffage to fee, that, according to the doctrine of Mr. Hunter, (and may I add myfelf?) Dr. Haygarth fhould have written, "from a *morbid*," inftead of 'from the venereal poifon." Having already fhewn my refpect for this ingenious writer, by adopting his language, and availing myfelf of his facts, (fee "Morbid Poifons," pages 49, 51, and 51,) he will not be offended that I differ from him on this occafion.

Dr. Haygarth concludes his paper in the following words:— "Thus, in three fucceffive cafes, the venereal poifon applied to to the finger, produced ſordid, ſpongy, painful, phagedænic ulcers, inflamed lymphatic abſorbents and glands, and manifeſt ſymptoms of a low nervous fever." In anſwer to this, it ſhould be recollected, that the ſpongy or phagedænic ulcer is not leſs remote than the low nervous fever from the character of the primary venereal ulcer or chancre; and alſo that fever has been detected in moſt, if not all, thoſe anomalous morbid poiſons which have been confounded with the venereal. Nor does this depend on the nature of the part affected, for in the following paſſage, which I tranſcribe from "Morbid Poiſons," to ſave you the trouble of referring to the work, the four laſt-mentioned caſes were ſeated on the penis.

"If we examine carefully the accounts of all the tooth caſes, we ſhall find moſt, if not all of them, attended with fever during their whole ſtage. Sir W. Watſon and Dr. Lettſom mention it particularly in the caſes they relate, and Mr. Hunter in moſt of thoſe, the whole progreſs of which fell under his inſpection. Mr. French particularizes fever in the caſe of ſloughing of the glands before alluded to. I have remarked it in the caſe of ſloughing phagedæna. The ſame may be traced in the caſe related in Edin. Medic. Eſſays; and Turner deſcribes his patient as delirious with fever and a miliary eruption *."

Dr. Haygarth's firſt caſe was followed by ſecondary ſymptoms, and "a formidable acceſſion of fever." Now, though the ſecondary ſymptoms from venereal virus are uſually attended with fever, yet it is never ſuch as would admit of the above deſcription from a writer of that gentleman's accuracy. It is moſt commonly ſo ſlight as to be overlooked altogether, and when greateſt, never exceeds the common ſymptoms of mild hectic, recurring for a few days at regular periods, afterwards remitting, and then recurring with its former apparent regularity.

I am aware it may be urged, that if theſe diſeaſes all yield to mercury, it is of little conſequence whether they are venereal or no. But it is hardly neceſſary to remind you, that though we might by analogy expect the ſame relief in all thoſe morbid poiſons which, like the venereal, do not leave the conſtitution after a critical fever, yet experience convinces us we are too often diſappointed: beſides this, the reputation of the ſex, or the tranquillity of the *inrupta copula*, is often involved in the queſtion. Need I add, that had ſome writers of the preſent day been more accurate in the deſcription of local ſymptoms, much of that ACIDITY and gall might have been ſpared, which

* See "Morbid Poiſons," page 146. Johnſon, 1796.

have been expended in the controversy concerning new remedies.

After all, we are much obliged to Dr. Haygarth for his three valuable cases, and the more so, as the local appearances are so accurately described. Be so good as to acquaint him with my objection to his inferences, in any manner you please; but if the Editors of the Journal can find room, it would be desirable to make them as public as Dr. Haygarth's remarks.

I remain, my dear Sir,

Your sincere friend,

*Madeira, Jan.* 14, 1801. JOSEPH ADAMS.

